# US seniors' intention to vaccinate against RSV in fall and winter 2023–2024

**DOI:** 10.1093/haschl/qxae003

**Published:** 2024-01-19

**Authors:** Simon F Haeder

**Affiliations:** Department of Health Policy and Management, School of Public Health, Texas A&M University, College Station, TX 77843, United States

**Keywords:** RSV, seniors, vaccines, hesitancy

## Abstract

In the fall and winter of 2023–2024, the United States may experience a “tripledemic” of COVID-19, influenza, and respiratory syncytial virus (RSV) that may lead to 100 000 deaths. Seniors will be disproportionally affected. The newly released RSV vaccines for those age 60 years and over may reduce the number of deaths for the expected 6000 to 10 000 seniors expected to die from RSV alone. Using a large national survey, we queried Americans over age 60 about their RSV vaccination status and their intention to vaccinate this fall and winter. We found that 9% of seniors had already been vaccinated. Of the remaining unvaccinated, 42% indicated their intent to vaccinate. We found that those with higher levels of concerns for the disease, higher levels of self-assessed risk, believing that vaccines were safe and important, higher levels of trust in health institutions, and men were more likely to seek out vaccinations. Vaccine-hesitant respondents listed a lack of necessity, concerns about side effects and safety, and a lack of information as primary reasons. The large number of unvaccinated seniors will likely lead to an excessive number of hospitalizations and deaths as well as augmented social costs. Evidence-based mitigation measures tailored to seniors' concerns should be implemented immediately.

## Introduction

Respiratory syncytial virus (RSV) poses a significant health risk to populations worldwide but particularly to the very young and the old, and in those with chronic conditions.^1^ Older adults above age 60 years are particularly affected. Every year between 60 000 and 160 000 US seniors are hospitalized, with 6000 to 10 000 ultimately dying of the infection.^2^ However, recently, 2 vaccines became available for older adults that hold the potential to substantially reduce these numbers.^3^ The vaccines are 83% to 89% effective in preventing lung infections.^4,5^ However, there are reasons to be skeptical about how widespread vaccinations among US seniors will be in the fall and winter 2023–2024. For one, the Centers for Disease Control and Prevention (CDC) did not make an outright recommendation for the vaccines,^3^ a decision that has been criticized by some.^6^ This ambivalence has been mirrored by various other important sources of advice for older Americans including the AARP,^7^ the American Medical Association,^8^ the American Hospital Association,^9^ and the American Pharmacists Association.^10^ At the same time, vaccine hesitancy has been growing across the globe,^11,12^ particularly in the wake of COVID-19.^13-20^ Growing vaccine hesitancy is concerning not only because it leads to higher sickness, hospitalizations, and deaths among vaccine-hesitant groups but also because of the substantial positive externalities involved with vaccinations, particularly for marginalized communities and vulnerable populations.^21-23^ Reducing RSV-related illness among seniors is a crucial goal because of the expected “tripledemic” of COVID-19, influenza, and RSV that may lead to 100 000 deaths in the United States this fall and winter.^24^ However, vaccination intentions among US seniors against RSV remain underexplored so far.

## Material and methods

### Data

To learn more about the intentions to vaccinate against RSV for Americans over 60, we fielded a nationally representative survey on September 27 and September 28, 2023, using Lucid. Lucid has been used extensively in this type of research because it provides high-quality survey data and the data have been validated against true probability samples.^25^ Lucid relies on quota sampling by age, educational attainment, household income, race and ethnicity, and partisan identification. Respondents are selected into the survey via a double opt-in procedure, first joining the Lucid panel and subsequently agreeing to participate in the survey.^25^ Lucid was compensated $1.50 per completed response. For additional details, please refer to the Appendix Exhibits 1 through 4.

Of the 7706 respondents who opted into the survey, 7360 (96%) completed the consent form and ultimately 5035 respondents completed the survey (68%). The main source of respondent attrition were 2 attention checks utilized to ensure data quality (31%). Less than 1% of respondents failed to pass the reCAPTCHA verification. A total of 1345 respondents were aged 60 and older. Data were weighted on age, gender, race, income, and education based on the Current Population Survey. The study received approval from the institutional review boards at the appropriate institutions.

### Measures

#### Dependent variables

All respondents over 60 were asked a number of questions related to RSV and the RSV vaccine. The 2 major outcomes of interest were whether respondents had already received the RSV vaccine or, if not, whether they intended to get vaccinated this fall and winter. Those respondents who indicated that they had no plans to get vaccinated also received a list of 12 distinct reasons for their hesitation based on previous literature.^5,17,20^ This list included lack of insurance coverage, lack of resources, lack of information, concerns about vaccine safety, concerns about vaccine side effects, concerns about vaccine effectiveness, whether they thought vaccines were not important, whether they saw no need for the vaccine, whether the process was too complicated, whether they lacked time, whether they already had the disease, and whether it was against their religious beliefs. See Appendix Exhibit 3 and 4 for detailed wording.

#### Independent variables

Respondents were also asked various questions commonly asked in assessments of vaccination intentions. These questions included their self-assessed level of concern about RSV using a 5-point scale from “Not at all” to “A great deal.”^17^ We also asked respondents to self-assess their personal risk of getting RSV in comparison to others on a 5-point scale from “much higher” to “much lower.”^26^ We expect that those with higher levels of self-assessed concerns and those with higher levels of self-assessed personal risk will be more likely to seek out vaccinations. We also queried respondents whether they had experienced RSV previously. We expect that previous infections will positively predict intentions to vaccinate.^26^ We also included standard measures asking respondents whether they thought that vaccines were safe, effective, and important (all 4-point scales) with the expectation that respondents scoring higher on these measures will be more likely to seek out vaccinations.^17,27^

Due to the increasing politicization and polarization of vaccinations, we also included measures to assess whether respondents had voted for President Trump in 2020 and whether they considered themselves to be liberal (combining both “extreme liberals” and “liberals”) or conservative (combining both “extreme conservatives” and “conservatives”). We expect that Trump voters and conservatives will be less likely to seek out vaccinations, with the opposite effect for liberals. We note that we re-estimated all models replacing ideology with partisanship with analogous results (see Appendix Exhibit 7). We also expect that respondents who are more religiously active (5-point scale) will be more hesitant to get vaccinated.^28^ Trust in health institutions has also been shown to be an important correlate of vaccine hesitancy.^17^ We measured trust as a 13-point index combining standard individual trust measures for the CDC, the National Institutes of Health, the Food and Drug Administration, and medical doctors (all 4-point scales from “no confidence at all” to “a great deal”; Cronbach's alpha: .86). Additional control variables accounted for respondents' race and ethnicity, gender, age, income, education, and insurance status (Medicare, Medicaid, individual market, employer-sponsored insurance, uninsured).^17,27^

### Methodological approach

First, we derived unadjusted estimates for seniors who either were already vaccinated or expressed their intent to get vaccinated. Second, we relied on multivariate logit regression utilizing survey weights to assess previous vaccination as well as intention to vaccinate against RSV because our outcome variables described above are dichotomous. Due to a lack of interpretability of logit coefficients, we then make use of average marginal effects (AMEs) to assess substantive and statistical significance of the various variables of interest.^29,30^ We also present odds ratios. Across these analyses, we considered a *P* value lower than .05 as statistically significant. Third, we also we derived unadjusted estimates for the various underlying reasons that seniors provided for not seeking out vaccinations.

## Results

**Figure 1.**
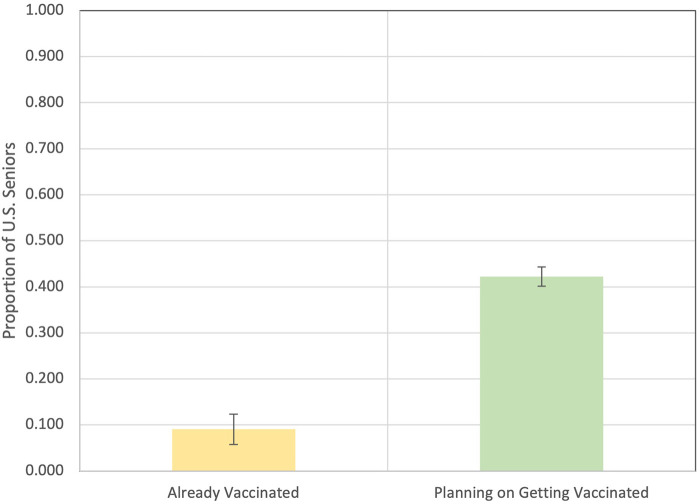
Proportion of respondents who indicated that they are already vaccinated against RSV, are planning on getting vaccinated, and a combination of both. Weighted unadjusted estimates based on survey sample. Abbreviation: RSV, respiratory syncytial virus.

Overall (Figure 1), 9.1% (95% CI, 7.3%–11.2%) of respondents indicated that they had already been vaccinated against RSV. Moreover, out of those who had not been vaccinated, 42.2% (38.9%–45.7%) indicated their intent to do so “this fall and winter.” This combines for a total of 47.5% (44.2%–50.8%) of respondents (see also Appendix Exhibit 5).

### Correlates of vaccination and intentions to vaccinate

The logit models presented in Table 1 further parse out the correlates of previous vaccination as well as intention to vaccinate (see also Appendix Exhibits 6 and 8). Included in Table 1 are also AMEs where their *P* value did not exceed .05 as well as odds ratios. Assessing the group of respondents who had already received the RSV vaccine, we found that women were less likely to already have received the vaccine (AME: −0.046; *P* = .014). In addition, we found that those obtaining coverage in the individual market were less likely to be vaccinated (−0.093; *P* < .001). Focusing on the respondents who had not yet received the vaccine, we found various correlates of intention to vaccinate. As expected, those with higher levels of concern for the disease (0.098; *P* < .001) and those with higher self-assessed risks for the disease (0.092; *P* < .001) were more likely to signal their intent to get vaccinated. The same held for those with the belief that vaccines were safe (0.105; *P* = .003) and important (0.094; *P* = .013), and those with higher levels of trust in health institutions (0.021; *P* = .001). We note that we did not find any statistically significant results for the political variables included. This remained the case when we re-estimated the models replacing presidential vote choice and ideology with Democratic and Republican partisanship (see Appendix Exhibit 7). However, while the statistically significant findings from our primary models remained in the re-estimated versions, we also found that those with higher levels of religious activity (−0.020; *P* = .045) were more hesitant to vaccinate.

**Table 1.** Results for logit regressions for intention to vaccinate against RSV.

	(1)			(2)		
	Already vaccinated			Planning on getting vaccinated		
Variables	Logit coefficient (*n* = 1294)	Odds ratio	AME	Logit coefficient (*n* = 1182)	Odds ratio	AME
Trump voter	0.330	1.391		−0.006	0.994	
	(.343)	(.343)		(.979)	(.979)	
Liberal	0.094	1.099		0.257	1.293	
	(.747)	(.747)		(.290)	(.290)	
Conservative	−0.094	0.910		−0.111	0.895	
	(.774)	(.774)		(.641)	(.641)	
Religiosity (low to high)	−0.057	0.944		−0.083	0.920	
	(.518)	(.518)		(.222)	(.222)	
Female	−0.636**	0.529**	−0.046	−0.319*	0.727*	
	(.014)	(.014)	0.014	(.088)	(.088)	
Vaccines are safe	1.111***	3.037***		0.692***	1.997***	0.105
	(.009)	(.009)		(.004)	(.004)	0.003
Vaccines are effective	−0.527	0.590		0.035	1.036	
	(.135)	(.135)		(.895)	(.895)	
Vaccines are important	0.226	1.254		0.616**	1.852**	0.094
	(.568)	(.568)		(.017)	(.017)	0.013
Concern about disease (low to high)	0.292**	1.339**		0.643****	1.903****	0.098
	(.034)	(.034)		(.000)	(.000)	0.000
Risk for disease (high to low)	−0.065	0.937		−0.625****	0.535****	−0.092
	(.696)	(.696)		(.000)	(.000)	0.000
Previously ill with disease	1.257**	3.515**		0.596	1.814	
	(.014)	(.014)		(.228)	(.228)	
Trust in health institutions (low to high)	−0.034	0.966		0.136****	1.146****	0.021
	(.617)	(.617)		(.001)	(.001)	0.001
Medicare	−0.129	0.879		0.180	1.197	
	(.801)	(.801)		(.703)	(.703)	
Medicaid	−0.446	0.640		0.443	1.557	
	(.522)	(.522)		(.484)	(.484)	
Individual market	−3.706***	0.025***	−0.093	0.124	1.132	
	(.001)	(.001)	0.000	(.823)	(.823)	
Uninsured	−0.300	0.741		0.016	1.016	
	(.771)	(.771)		(.981)	(.981)	
Employer-sponsored insurance	−0.412	0.662		−0.099	0.905	
	(.478)	(.478)		(.844)	(.844)	
Non-Hispanic White	−0.180	0.835		0.377	1.457	
	(.782)	(.782)		(.404)	(.404)	
Non-Hispanic Black	−0.543	0.581		0.638	1.893	
	(.546)	(.546)		(.292)	(.292)	
Non-Hispanic Asian	0.193	1.212		0.231	1.260	
	(.831)	(.831)		(.742)	(.742)	
Hispanic	0.145	1.156		−0.586	0.557	
	(.867)	(.867)		(.388)	(.388)	
Income	0.072	1.075		0.081	1.084	
	(.449)	(.449)		(.224)	(.224)	
Education	0.023	1.023		0.163	1.176	
	(.896)	(.896)		(.188)	(.188)	
Age	−0.135**	0.874**		−0.066	0.936	
	(.045)	(.045)		(.214)	(.214)	
Age^2^	0.001*	1.001*		0.001	1.001	
	(.088)	(.088)		(.252)	(.252)	
Constant	−0.529	0.589		−4.718**	0.009**	
	(.817)	(.817)		(.011)	(.011)	
Average prediction, Pr(y | base)			0.090			0.423
Observations	1294	1294		1182	1182	

Abbreviations: AME, average marginal effect; RSV, respiratory syncytial virus.

Results based on logit regression with survey weights. *P* values in parentheses. ****P < .001, ***P < .01, **P < .05, *P < .10.

### Reasons provided for hesitancy to vaccinate against RSV

As noted above, we also asked respondents who had indicated that they had no intention to get vaccinated against RSV for the underlying reasons of their hesitation (Table 2). The primary drivers of hesitation included not seeing the necessity for the vaccine (25.0%; 22.3%–28.0%), a lack of information (23.8%; 21.1%–26.8%), concerns about side effects (22.3%; 19.7%–25.1%), and concerns about vaccine safety (12.8%; 10.8%–15.2%). We note that resource limitations like time and finances as well as religious beliefs were not important contributors.

**Table 2.** Reasons provided for hesitancy to vaccinate against RSV.

		95% CI	
Reason	Estimate	Lower	Upper
Do not think I need the vaccine	0.250	0.223	0.280
Do not think I have enough information	0.238	0.211	0.268
Worried about the side effects of the vaccine	0.223	0.197	0.251
Do not think the vaccines are safe	0.128	0.108	0.152
Do not think the vaccines work	0.072	0.057	0.090
Do not think the vaccines are important	0.041	0.030	0.055
Do not have the financial resources/too expensive	0.018	0.012	0.029
Do not have health insurance	0.009	0.004	0.017
Not in line with my religious beliefs	0.006	0.003	0.013
Already had the disease	0.006	0.003	0.013
Do not have the time	0.004	0.002	0.009
Process is too complicated	0.003	0.001	0.008

Abbreviation: RSV, respiratory syncytial virus.

Weighted unadjusted estimates based on survey sample.

## Discussion and conclusion

Experts expect that the United States will experience a substantial number of individuals falling ill from COVID-19, influenza, and RSV this fall and winter.^24^ Seniors will be disproportionately affected by all 3 vaccine-preventable diseases. Our findings here indicate that only a minority of those above age 60 will seek out vaccinations against RSV, a disease that claims the lives of 6000 to 10 000 seniors every year.^2^ These numbers are also much lower than comparable numbers for young children.^5^ Our findings indicate that intentions to vaccinate were lower among women, those who do not deem vaccines safe or important, those with lower levels of concern or self-assessed risk for RSV, and those with lower levels of trust in health institutions. As a result of the low levels of intended vaccinations, it seems likely that the United States will experience a substantial impact on its health care system dealing with a large number of individuals sick from diseases that could have been prevented or mitigated by vaccinations. Individuals who fail to get vaccinated, as well as those who medically cannot receive the vaccine, will be disproportionately affected. However, societal costs will likely be large and affect society as a whole.

Our study comes with several limitations. First, we utilized an online panel that is representative of the United States nationally along important dimensions to generate the data for our analyses. This is common practice today, and the panel we used is reputable, validated, and frequently employed for this type of work.^25^ We further improved data quality by using a reCAPTCHA verification procedure and 2 attention checks. Second, our data are cross-sectional in nature and thus, by definition, subject to several limitations. Third, while RSV receives media attention, it is not as well known as other vaccine-preventable diseases like influenza and COVID-19. Not all seniors may be fully aware of their risks or previous infections, for example. Last, we restricted our analyses to Americans aged 60 and older, the target group for the new RSV vaccine. As noted above, our data are nationally representative of the US adult population. However, there are no a priori reasons to assume that, accounting for our large sample, our selection would introduce potential bias into our analyses. We also utilized various survey weights to account for potential differences and found consistent results.

Given that a majority of seniors are unlikely to seek out RSV vaccinations this year, questions emerge how policymakers can respond. Policymakers ought to respond to these findings by implementing evidence-based policies that increase vaccination rates in the immediate as well as long-term future.^23^ Based on the findings here, these efforts should focus on highlighting the safety and importance of vaccines as well as the potential risks to individuals who fail to vaccinate.^31,32^ Moreover, substantial efforts are needed to counter false and misleading information about both the disease as well as the vaccines.^31,32^ An important role also falls to health care providers, who tend to be the most trusted source of medical advice for most individuals.^31,32^ Moreover, given the consistent findings about increased vaccine hesitancy in women, approaches particularly tailored to women ought to be developed. Ultimately, policymakers and society would be well served if effective measures to lower vaccine hesitancy across the board were to be implemented.

## Supplementary Material

qxae003_Supplementary_Data
