# Supplementary material for: US seniors' intention to vaccinate against RSV in fall and winter 2023–2024
Source: Health Aff Sch. 2024 Jan 19;2(2):qxae003. doi: 10.1093/haschl/qxae003 (PMC10986196; doi:10.1093/haschl/qxae003)
Supplement: qxae003_Supplementary_Data [file qxae003_Supplementary_Data.zip › Appendix R1 v2.docx]

**Appendix**

**Appendix: Exhibit 1:** Distribution of Weights

**Appendix: Exhibit 2a:** Comparison of Raw and Weighted Qualtrics Data to National Benchmarks, U.S. Adults Population

| **Variable** | **Survey Data**  **(Raw)** | **Survey Data**  **(Weighted)** | **Benchmark** | **Benchmark Source** |
| --- | --- | --- | --- | --- |
|  |  |  |  |  |
| Female | 51% | 51% | 51% | CPS |
| College Degree | 40% | 33% | 31% | CPS |
| Black | 10% | 12% | 13% | CPS |
| White | 70% | 64% | 62% | CPS |
| Hispanic | 11% | 16% | 18% | CPS |
| Democrat | 34% | 34% | 34% | ANES (Wgt.) |
| Republican | 29% | 30% | 28% | ANES (Wgt.) |
| Mean Age | 45 | 46 | 47 | ANES (Wgt.) |
| Median Income | $35 to $49,999 | $50 to 74,999 | $55 – 59,999 | ANES (Wgt.) |

Note: Comparison of the data to known population benchmarks. CPS = Current Population Survey. ANES = American National Election Study. Preference is given to CPS considering its sample size and representativeness, but make use of weighted ANES data whenever it was not possible to use CPS (i.e. CPS does not ask questions about Party ID). Weights in column two adjust for gender, education, race, age, and income. N (Survey Data) = 5,035

**Appendix: Exhibit 2b:** Comparison of Raw and Weighted Qualtrics Data to National Benchmarks, U.S. Seniors

| **Variable** | **Survey Data**  **(Raw)** | **Survey Data**  **(Weighted)** | **Benchmark** | **Benchmark Source** |
| --- | --- | --- | --- | --- |
|  |  |  |  |  |
| Female | 52% | 54% | 55% | CPS |
| College Degree | 44% | 39% | 29% | CPS |
| Black | 7% | 9% | 9% | CPS |
| White | 83% | 79% | 77% | CPS |
| Hispanic | 4% | 7% | 8% | CPS |
| Median Income | $35 to 49,999 | $50 to 74,999 | $50 to 74,999 | ANES (Wgt.) |

Note: Comparison of the data to known population benchmarks. CPS = Current Population Survey. ANES = American National Election Study. Weights in column two adjust for gender, education, race, age, and income. N (Survey Data) = 1,341

**Appendix: Exhibit 3: Survey Question for RSV**

You may have heard that there is new vaccine against Respiratory Syncytial Virus (RSV) for those over age 60. We want to learn more about how individuals like you think about this vaccine.

How concerned are you about you getting RSV?

o Not at all (1)

o A little (2)

o A moderate amount (3)

o A lot (4)

o A great deal (5)

Compared to others, do you think your risk of getting RSV is ...

o Much higher (1)

o Somewhat higher (2)

o About the same (3)

o Somewhat lower (4)

o Much lower (5)

To your knowledge, have you ever had RSV?

o Yes (1)

o No (2)

Have you already gotten vaccinated with the RSV vaccine?

o Yes (1)

o No (2)

Are you planning on getting vaccinated against RSV this fall or winter?

o Yes (1)

o No (2)

Why aren't you planning to get vaccinated against RSV this fall or winter? Mark all that apply.

▢ Do not have health insurance (1)

▢ Do not have the financial resources/Too expensive (2)

▢ Do not think I have enough information on vaccines (3)

▢ Do not think the vaccines are safe (4)

▢ Worried about the side effects of the vaccine (5)

▢ Do not think the vaccines work (6)

▢ Do not think the vaccines are important (7)

▢ Do not think I need the vaccine (8)

▢ Process is too complicated (9)

▢ Do not have the time (10)

▢ Already had RSV (11)

▢ Not in line with my religious beliefs (12)

**Appendix: Exhibit 4: Survey Question about Vaccines**

In general, do you believe that vaccines are safe?

o Definitely not (1)

o Probably not (2)

o Probably yes (3)

o Definitely yes (4)

In general, do you believe vaccines are effective?

o Definitely not (1)

o Probably not (2)

o Probably yes (3)

o Definitely yes (4)

In general, do you believe that vaccines are important?

o Definitely not (1)

o Probably not (2)

o Probably yes (3)

o Definitely yes (4)

**Appendix: Exhibit 5:** Proportion of Respondents Who Indicated That They Are Already Vaccinated against RSV, Are Planning on Getting Vaccinated, and a Combination of Both

Notes: Weighted unadjusted estimates based on survey sample.

**Appendix: Exhibit 6:** Results for Logit Regressions for Intention to Vaccinate against RSV

|  | (1) | | | (2) | | | (3) | | |
| --- | --- | --- | --- | --- | --- | --- | --- | --- | --- |
|  | Already Vaccinated | | | Planning on Getting Vaccinated | | | Combined | | |
| VARIABLES | Logit Coefficient | **Odds Ratio** | AME | Logit Coefficient | **Odds Ratio** | AME | Logit Coefficient | **Odds Ratio** | AME |
|  | **N=**1,294 | | | **N=**1,182 | | | **N=**1,294 | | |
|  |  |  |  |  |  |  |  |  |  |
| Trump Voter | 0.330 | 1.391 |  | -0.006 | 0.994 |  | 0.013 | 1.013 |  |
|  | (0.343) | (0.343) |  | (0.979) | (0.979) |  | (0.955) | (0.955) |  |
| Liberal | 0.094 | 1.099 |  | 0.257 | 1.293 |  | 0.250 | 1.284 |  |
|  | (0.747) | (0.747) |  | (0.290) | (0.290) |  | (0.287) | (0.287) |  |
| Conservative | -0.094 | 0.910 |  | -0.111 | 0.895 |  | -0.118 | 0.888 |  |
|  | (0.774) | (0.774) |  | (0.641) | (0.641) |  | (0.591) | (0.591) |  |
| Religiosity | -0.057 | 0.944 |  | -0.083 | 0.920 |  | -0.103 | 0.902 |  |
|  | (0.518) | (0.518) |  | (0.222) | (0.222) |  | (0.113) | (0.113) |  |
| Female | -0.636* | 0.529* | -0.046 | -0.319# | 0.727# |  | -0.403* | 0.669* | -0.063 |
|  | (0.014) | (0.014) | 0.014 | (0.088) | (0.088) |  | (0.024) | (0.024) | 0.023 |
| Vaccines are safe | 1.111** | 3.037** |  | 0.692** | 1.997** | 0.105 | 0.878*** | 2.407*** | 0.135 |
|  | (0.009) | (0.009) |  | (0.004) | (0.004) | 0.003 | (0.000) | (0.000) | 0.000 |
| Vaccines are effective | -0.527 | 0.590 |  | 0.035 | 1.036 |  | -0.135 | 0.874 |  |
|  | (0.135) | (0.135) |  | (0.895) | (0.895) |  | (0.591) | (0.591) |  |
| Vaccines are important | 0.226 | 1.254 |  | 0.616* | 1.852* | 0.094 | 0.615* | 1.850* | 0.096 |
|  | (0.568) | (0.568) |  | (0.017) | (0.017) | 0.013 | (0.012) | (0.012) | 0.008 |
| Concern about disease | 0.292* | 1.339* |  | 0.643*** | 1.903*** | 0.098 | 0.624*** | 1.866*** | 0.097 |
|  | (0.034) | (0.034) |  | (0.000) | (0.000) | 0.000 | (0.000) | (0.000) | 0.000 |
| Risk for disease | -0.065 | 0.937 |  | -0.625*** | 0.535*** | -0.092 | -0.533*** | 0.587*** | -0.083 |
|  | (0.696) | (0.696) |  | (0.000) | (0.000) | 0.000 | (0.000) | (0.000) | 0.000 |
| Previously ill with disease | 1.257* | 3.515* |  | 0.596 | 1.814 |  | 0.778# | 2.176# |  |
|  | (0.014) | (0.014) |  | (0.228) | (0.228) |  | (0.067) | (0.067) |  |
| Trust in Health Institutions | -0.034 | 0.966 |  | 0.136*** | 1.146*** | 0.021 | 0.107** | 1.112** | 0.017 |
|  | (0.617) | (0.617) |  | (0.001) | (0.001) | 0.001 | (0.006) | (0.006) | 0.005 |
| Medicare | -0.129 | 0.879 |  | 0.180 | 1.197 |  | 0.096 | 1.100 |  |
|  | (0.801) | (0.801) |  | (0.703) | (0.703) |  | (0.813) | (0.813) |  |
| Medicaid | -0.446 | 0.640 |  | 0.443 | 1.557 |  | 0.217 | 1.242 |  |
|  | (0.522) | (0.522) |  | (0.484) | (0.484) |  | (0.691) | (0.691) |  |
| Individual Market | -3.706** | 0.025** | -0.093 | 0.124 | 1.132 |  | -0.254 | 0.775 |  |
|  | (0.001) | (0.001) | 0.000 | (0.823) | (0.823) |  | (0.608) | (0.608) |  |
| Uninsured | -0.300 | 0.741 |  | 0.016 | 1.016 |  | -0.322 | 0.725 |  |
|  | (0.771) | (0.771) |  | (0.981) | (0.981) |  | (0.593) | (0.593) |  |
| Employer-Sponsored Insurance | -0.412 | 0.662 |  | -0.099 | 0.905 |  | -0.144 | 0.866 |  |
|  | (0.478) | (0.478) |  | (0.844) | (0.844) |  | (0.743) | (0.743) |  |
| Non-Hispanic White | -0.180 | 0.835 |  | 0.377 | 1.457 |  | 0.319 | 1.375 |  |
|  | (0.782) | (0.782) |  | (0.404) | (0.404) |  | (0.426) | (0.426) |  |
| Non-Hispanic Black | -0.543 | 0.581 |  | 0.638 | 1.893 |  | 0.653 | 1.921 |  |
|  | (0.546) | (0.546) |  | (0.292) | (0.292) |  | (0.243) | (0.243) |  |
| Non-Hispanic Asian | 0.193 | 1.212 |  | 0.231 | 1.260 |  | 0.109 | 1.115 |  |
|  | (0.831) | (0.831) |  | (0.742) | (0.742) |  | (0.865) | (0.865) |  |
| Hispanic | 0.145 | 1.156 |  | -0.586 | 0.557 |  | -0.122 | 0.886 |  |
|  | (0.867) | (0.867) |  | (0.388) | (0.388) |  | (0.840) | (0.840) |  |
| Income | 0.072 | 1.075 |  | 0.081 | 1.084 |  | 0.086 | 1.090 |  |
|  | (0.449) | (0.449) |  | (0.224) | (0.224) |  | (0.179) | (0.179) |  |
| Education | 0.023 | 1.023 |  | 0.163 | 1.176 |  | 0.135 | 1.145 |  |
|  | (0.896) | (0.896) |  | (0.188) | (0.188) |  | (0.238) | (0.238) |  |
| Age | -0.135* | 0.874* |  | -0.066 | 0.936 |  | -0.094# | 0.910# |  |
|  | (0.045) | (0.045) |  | (0.214) | (0.214) |  | (0.080) | (0.080) |  |
| Age^2^ | 0.001# | 1.001# |  | 0.001 | 1.001 |  | 0.001 | 1.001 |  |
|  | (0.088) | (0.088) |  | (0.252) | (0.252) |  | (0.119) | (0.119) |  |
| Constant | -0.529 | 0.589 |  | -4.718* | 0.009* |  | -3.143# | 0.043# |  |
|  | (0.817) | (0.817) |  | (0.011) | (0.011) |  | (0.097) | (0.097) |  |
| Average Prediction (Pr(y\|base)) |  |  | 0.090 |  |  | 0.423 |  |  | 0.475 |
| Observations | 1,294 | 1,294 |  | 1,182 | 1,182 |  | 1,294 | 1,294 |  |

**Appendix: Exhibit 7:** Results for Logit Regressions for Intention to Vaccinate against RSV, Alternative Specification

|  | (1) | (2) | (3) | (1) | (2) | (3) |
| --- | --- | --- | --- | --- | --- | --- |
|  | Already Vaccinated | | Planning on Getting Vaccinated | | Combined | |
| VARIABLES | Coefficient | AME | Coefficient | Coefficient | AME | Coefficient |
|  |  |  |  |  |  |  |
| Democrat | 0.177 |  | 0.234 |  | 0.257 |  |
|  | (0.585) |  | (0.292) |  | (0.215) |  |
| Republican | 0.197 |  | 0.131 |  | 0.170 |  |
|  | (0.601) |  | (0.588) |  | (0.458) |  |
| Religiosity | -0.053 |  | -0.109 |  | -0.130* | -0.020 |
|  | (0.580) |  | (0.102) |  | (0.046) | 0.045 |
| Female | -0.662** | -0.048 | -0.310# |  | -0.392* | -0.062 |
|  | (0.010) | 0.010 | (0.096) |  | (0.027) | 0.026 |
| Vaccines are safe | 1.098** | 0.113 | 0.707** | 0.108 | 0.889*** | 0.137 |
|  | (0.008) | 0.046 | (0.003) | 0.003 | (0.000) | 0.000 |
| Vaccines are effective | -0.528 |  | 0.033 |  | -0.144 |  |
|  | (0.129) |  | (0.903) |  | (0.576) |  |
| Vaccines are important | 0.233 |  | 0.579* | 0.088 | 0.590* | 0.092 |
|  | (0.547) |  | (0.026) | 0.022 | (0.016) | 0.012 |
| Concern about disease | 0.291* |  | 0.655*** | 0.100 | 0.635*** | 0.099 |
|  | (0.035) |  | (0.000) | 0.000 | (0.000) | 0.000 |
| Risk for disease | -0.066 |  | -0.621*** | -0.091 | -0.527*** | -0.082 |
|  | (0.695) |  | (0.000) | 0.000 | (0.000) | 0.000 |
| Previously ill with disease | 1.237* |  | 0.605 |  | 0.777# |  |
|  | (0.015) |  | (0.225) |  | (0.068) |  |
| Trust in Health Institutions | -0.048 |  | 0.148*** | 0.022 | 0.118** | 0.018 |
|  | (0.453) |  | (0.000) | 0.000 | (0.002) | 0.002 |
| Medicare | -0.129 |  | 0.190 |  | 0.096 |  |
|  | (0.803) |  | (0.687) |  | (0.811) |  |
| Medicaid | -0.475 |  | 0.505 |  | 0.276 |  |
|  | (0.505) |  | (0.432) |  | (0.617) |  |
| Individual Market | -3.736** |  | 0.073 |  | -0.311 |  |
|  | (0.001) |  | (0.894) |  | (0.526) |  |
| Uninsured | -0.282 |  | 0.055 |  | -0.299 |  |
|  | (0.787) |  | (0.935) |  | (0.620) |  |
| Employer-Sponsored Insurance | -0.376 |  | -0.058 |  | -0.104 |  |
|  | (0.518) |  | (0.909) |  | (0.813) |  |
| Non-Hispanic White | -0.160 |  | 0.328 |  | 0.272 |  |
|  | (0.803) |  | (0.477) |  | (0.508) |  |
| Non-Hispanic Black | -0.621 |  | 0.586 |  | 0.606 |  |
|  | (0.488) |  | (0.340) |  | (0.284) |  |
| Non-Hispanic Asian | 0.154 |  | 0.211 |  | 0.102 |  |
|  | (0.861) |  | (0.767) |  | (0.876) |  |
| Hispanic | 0.183 |  | -0.649 |  | -0.171 |  |
|  | (0.825) |  | (0.347) |  | (0.780) |  |
| Income | 0.081 |  | 0.082 |  | 0.087 |  |
|  | (0.389) |  | (0.219) |  | (0.166) |  |
| Education | 0.014 |  | 0.174 |  | 0.149 |  |
|  | (0.938) |  | (0.152) |  | (0.185) |  |
| Age | -0.145* |  | -0.068 |  | -0.098# |  |
|  | (0.034) |  | (0.194) |  | (0.070) |  |
| Age^2^ | 0.001# |  | 0.001 |  | 0.001 |  |
|  | (0.067) |  | (0.238) |  | (0.109) |  |
| Constant | -0.120 |  | -4.732** |  | -3.141# |  |
|  | (0.958) |  | (0.009) |  | (0.092) |  |
| Average Prediction (Pr(y\|base)) |  | 0.090 |  | 0.422 |  | 0.474 |
| Observations | 1,296 |  | 1,184 |  | 1,296 |  |

Notes: Results based on logit regression with survey weights. P-values in parentheses.

*** p<0.001, ** p<0.01, * p<0.05, # p<0.10

**Appendix: Exhibit 8:** Predicted Probabilities for Respondents Who Indicated That They Are Already Vaccinated against RSV, Are Planning on Getting Vaccinated, and a Combination of Both for Three Ideal Types
